# SENNA Inventory for the Assessment of Social and Emotional Skills in Public School Students in Brazil: Measuring Both Identity and Self-Efficacy

**DOI:** 10.3389/fpsyg.2021.716639

**Published:** 2021-11-25

**Authors:** Ricardo Primi, Daniel Santos, Oliver P. John, Filip De Fruyt

**Affiliations:** ^1^Post Graduate Program in Psychology, Universidade São Francisco, Campinas, Brazil; ^2^EduLab21, Ayrton Senna Institute, São Paulo, Brazil; ^3^Faculty of Economics, Administration and Accounting of Ribeirão Preto, University of São Paulo, Ribeirão Preto, Brazil; ^4^Department of Psychology and Institute of Personality and Social Research, University of California, Berkeley, Berkeley, CA, United States; ^5^Department of Developmental, Personality and Social Psychology, Ghent University, Ghent, Belgium

**Keywords:** 21st century skills, social-emotional skills, instrument development, Big Five, five-factor model, measurement invariance, exploratory structural equation modeling

## Abstract

Responding to the need for school-based, broadly applicable, low-cost, and brief assessments of socio-emotional skills, we describe the conceptual background and empirical development of the SENNA inventory and provide new psychometric information on its internal structure. Data were obtained through a computerized survey from 50,000 Brazilian students enrolled in public school grades 6 to 12, spread across the entire State of São Paulo. The SENNA inventory was designed to assess 18 particular skills (e.g., empathy, responsibility, tolerance of frustration, and social initiative), each operationalized by nine items that represent three types of items: three positively keyed trait-identity items, three negatively keyed identity items, and three (always positively keyed) self-efficacy items, totaling a set of 162 items. Results show that the 18 skill constructs empirically defined a higher-order structure that we interpret as the social-emotional Big Five, labeled as Engaging with Others, Amity, Self-Management, Emotional Regulation, and Open-Mindedness. The same five factors emerged whether we assessed the 18 skills with items representing (a) a trait-identity approach that emphasizes lived skills (what do I *typically* do?) or (b) a self-efficacy approach that emphasizes capability (*how well can I do that?*). Given that its target youth group is as young as 11 years old (grade 6), a population particularly prone to the response bias of acquiescence, SENNA is also equipped to correct for individual differences in acquiescence, which are shown to systematically bias results when not corrected.

## Introduction

Over the past two decades, education scientists and policy-makers showed an increased attention for the assessment and learning of Social-Emotional Skills, also called 21st century skills (from here onward abbreviated as SEMS) (Abrahams et al., 2019). This interest shift built on the notion that more traditional indicators of scholastic achievement, such as scores on math and reading, are not sufficient for a successful and happy life and for dealing with the challenges of today’s volatile, uncertain, complex, and ambiguous (VUCA) world^1^. In addition to content-specific knowledge and skills, students nowadays also need transferable skills such as collaboration and dealing with diversity, presentation skills, skills to regulate emotions, managerial and implementation skills, and a need to be open-minded, creative, and innovative, among others (De Fruyt, 2019). Hence, schools today pay considerable attention to the development of SEMS that they have been shown to affect a range of consequential outcomes, both in the short term and the long term (Taylor et al., 2017; Bertling and Alegre, 2018).

The increased attention to SEMS raised questions on their conceptual status, their underlying structure, and how SEMS develop normatively, driven by a complex interaction between biological factors and formal and informal learning experiences, including scholastic intervention (Kyllonen et al., 2014; Lipnevich et al., 2016; Abrahams et al., 2019). The reviews by Abrahams et al. (2019) further clarified that, in order to advance this field, we first need to converge on a taxonomy structuring SEMS, so we can design assessment instruments to examine pertinent research questions and support educators and teachers with formative and summative tools for assessing SEMS.

The present paper will review the conceptual background and construction of SENNA, an inventory designed to assess SEMS in Brazilian public education that is also useful for scientific and applied research. SENNA’s psychometric features will be examined in a sample of more than 50,000 students enrolled in grades 6 to 12 in public schools in the State of São Paulo. Little is known about the structure of socio-emotional variables in South America, especially in the public schools in Brazil that serve large numbers of underprivileged youth from poor and uneducated family backgrounds. Thus, the present research also provides important information about the applicability and generalizability of research from the Western, educated, rich, and industrialized countries (Henrich et al., 2010) that have so far dominated the SEMS research agenda.

### Defining and Structuring Social-Emotional Skills: The “Social-Emotional” Big Five

A Google search using the terms ‘social-emotional skills,’ ‘transferable skills’ or ‘21st century skills’ results in hundreds of hits referring to a plethora of different SEMS frameworks and taxonomies, with some advocating only a few and others proposing hundred or more skills^2^ (Abrahams et al., 2019). Table 1 provides an overview of some of the more comprehensive frameworks and their associated skills. Although there may be notable reasons why these frameworks differ in terms of the number and nature of specified SEMS, this mixture of models and diversity of vocabularies hampers an integrative and in-depth discussion on how to organize evidence-based learning of SEMS in education. The field further suffers from the jingle-jangle fallacy, where similarly named constructs across frameworks indicate different skills (jingle), whereas nearly identical skills are labeled differently by different researchers (jangle) (Olderbak and Wilhelm, 2020).

**TABLE 1:** Relationship of Big Five traits, functional aspects, and dimensions in major socio-emotional skills frameworks.

Big Five traits and facets (e.g., Soto and John, 2015, 2017)	Functional aspects	CASEL	CHICAGO consortium	OECD model	SENNA v2.0
**O: Open-mindedness** Creative imagination Intellectual curiosity Aesthetic sensitivity	Exploration system	*Self-awareness:* Identification and recognition of one’s own emotions		*Open-mindedness* Creativity Curiosity Tolerance	*Open-mindedness* Creative imagination Curiosity to learn Artistic interest
**C: Conscientiousness** Organization Productiveness Responsibility	Self-management system	*Self-management:* Persistence, goal setting, and motivation *Responsible decision making:* Evaluation and reflection, and personal and ethical responsibility.	Academic perseverance, learning strategies, academic behaviors	*Task performance* Responsibility Persistence Self-control Achievement-motivation	*Self-management* Determination Organization Focus Persistence Responsibility
**E: Extraversion** Sociability Assertiveness Energy level	Approach system	*Relationship skills:* Cooperation, help seeking and providing, and communication	Social skills	*Engaging with others* Sociability Assertiveness Energy	*Engaging with others* Social Initiative Assertiveness Enthusiasm
**A: Agreeableness** Compassion Respectfulness Trust	Belonging system	*Social awareness:* Empathy, respect for others, and perspective taking *Responsible decision making:* Evaluation and reflection, and personal and ethical responsibility.	Social skills	*Collaboration* Empathy Trust Co-operation	*Amity* Empathy Trust Respect Gratitude
**N: Emotional stability** (vs. neuroticism or negative emotionality) Anxiety Depression Emotional volatility	Coping system	*Self-awareness:* Sense of self-efficacy, and self-confidence. *Self-management:* Impulse control, stress management	Academic mindset	*Emotion regulation* Stress resistance Optimism Emotional control	*Negative-emotion regulation* Stress modulation Self-confidence Frustration tolerance

The challenge to bring order in the chaos of (often overlapping) skill terms closely resembles personality psychologists’ struggles to structure the hundreds of personality descriptive adjectives in the natural language into the Big Five personality taxonomy (John et al., 2008). Today, personality psychologists agree that five broad dimensions, namely Extraversion, Agreeableness, Conscientiousness, Emotional Stability (vs. Negative Emotionality), and Openness to Experiences, form the largest common denominator to describe personality differences observable in various age and cultural groups (McCrae and Terracciano, 2005; De Fruyt and Van Leeuwen, 2014; John, 2021). This encompassing empirical framework helped to solve the issues of overlap among constructs, and significantly advanced knowledge on how personality develops. A parallel trimming and structuring exercise is required for the field of SEMS.

During the past decade, the Organization of Economic Cooperation and Development (OECD) started an explicit study on the assessment of SEMS in a number of large cities around the world, as a complement of their Program of International Student Assessment^3^. John and De Fruyt (2015), serving in the technical advisory committee of this project, reviewed the SEMS literature at that time and concluded that there was an emerging consensus that the multitude of SEMS could be conceptually grouped in five broad skills categories, namely *Engaging with Others*, *Collaboration*, *Task Performance*, *Emotion Regulation, and Open-Mindedness*. Table 1 also includes more specific skills, such as persistence and empathy, and illustrates how they can be conceptually grouped into these broad dimensions. These five SEMS dimensions show strong conceptual similarities with the Big Five personality dimensions; hence we refer to them as the ‘Social-Emotional Big Five.’ This resemblance is not surprising, given that traits and cognitive abilities form constituting building blocks of competencies or SEMS (Hoekstra and Van Sluijs, 2003; Bartram, 2005).

The OECD (John and De Fruyt, 2015; Kankaraš and Suárez-Álvarez, 2019) and De Fruyt et al. (2015, p. 279) defined SEMS as “individual characteristics that: (a) originate in the reciprocal interaction between biological predispositions and environmental factors, (b) are manifested in consistent patterns of thoughts, feelings and behaviors, (c) continue to develop through formal and informal learning experiences, and (d) influence important socio-economic outcomes throughout the individual’s life.” This definition is broad enough to capture skills and their building blocks, underscoring their malleability across life and their consequential impact on outcomes that matter for the individual and society.

### Social-Emotional Skills in South America: Early Research in Brazil

In Brazil, interest in the development and assessment of SEMS has been steadily growing over the past 15 years to improve general welfare and prepare youth for upcoming challenges via public education and intervention programs. Challenges for the Brazilian public education systems have been numerous and urgent, with large and early school drop-out rates, poor results in PISA, complex social inequality challenges, high violence and crime rates, and considerable (youth) unemployment, amongst others (Miyamoto et al., 2014).

The Ayrton Senna Institute has played a pioneering role in raising awareness about the importance of SEMS in education and initiated and conducted a series of meetings and research activities to learn about SEMS assessment and their development. In 2013, the Ayrton Senna Institute collaborated with the OECD^4^ and the Education Secretariat of Rio de Janeiro to investigate the measurement of SEMS and describe their associations with various outcomes (Miyamoto et al., 2014; Santos and Primi, 2014). Leading the first empirical OECD study on SEMS in a developing country, Santos and Primi (2014) identified eight SEMS instruments with constructs that predicted consequential outcomes of education, were feasible to administer, assessed malleable constructs, and showed robust psychometric characteristics. In a large sample of middle school and high school students, Primi et al. (2016, 2019c) examined the items and scales of the Strengths and Difficulties Questionnaire (Goodman, 1997), the Self-Efficacy Questionnaire for Children (Muris, 2001), the Core Self-Evaluations (Judge et al., 2003), the Big Five Inventory (John et al., 2008), the Rosenberg Self-Esteem Scale (Rosenberg, 1965), the Nowicki-Strickland Locus of Control (Nowicki and Strickland, 1973) scale, the Big Five for Children (Barbaranelli et al., 2003), and the Grit Scale (Duckworth and Quinn, 2009) and found that they could all be mapped under the umbrella of the ‘Social-Emotional Big Five.’ Also, a sixth factor resulted from the factor analysis, reflecting beliefs about personal control versus low self-esteem, hopelessness, and feeling defeated, that connects with low Emotional Stability. This study provided the first empirical support for the conceptual classification proposed by John and De Fruyt (2015) and the OECD (2015) (Primi et al., 2016, 2019c).

Resulting from this work, a first set with 92 items was compiled (tentatively called SENNA 1.0) that was used in the OECD’s first pilot study on SEMS in Rio de Janeiro (Miyamoto et al., 2014; Santos and Primi, 2014; Primi et al., 2016). This first item set and data collection (*N* = 27,628) provided information at the broad Social-Emotional Big Five level and further helped to delineate requirements for a new broad SEMS measure that could be generally implemented in Brazilian education.

### Five Major Considerations for the Development of a Social-Emotional Skills Inventory for Public Education

First, it quickly became clear that a feasible large-scale assessment of SEMS in Brazilian public schools needed to be a *broadly applicable* survey, available at a *low cost*, requiring little *time* to administer (no more than 50 min of class time), and preferably useful for *self-description without assistance*. The assessment further had to result in *feedback* for individual students, but also reports at the level of classes (for teachers), schools (for directors) and educational districts/states (for policy makers). Besides these more formal necessities, there were also some critical content and technical requirements.

Second, to avoid a WEIRD-culture bias (Henrich et al., 2010) and to acknowledge the unique features of Brazilian culture and education, it was recommended to use an *emic and bottom-up approach* to the questionnaire constructions; the goal was to write and select items that would be age and culture appropriate for students in Brazilian public schools, covering the age range of grades 6 to 12 (i.e., typically age 11 to 18). These requirements suggested that a large new item set would be developed, and assessment procedures would be checked, always in close collaboration with students (through focus groups and pretesting), teachers, and various educational stakeholders.

Third, although there was some first evidence that the Social-Emotional Big Five was a useful framework (Primi et al., 2016, 2019c), educational stakeholders were interested to get information on students’ mastery of various more *specific skills*, such as responsibility, respect, or creativity, that could eventually be organized under the umbrella of the social-emotional five, as shown in Table 1. Information at the broad skill level is probably useful for directors and policy makers, but individual students and their teachers want to know the student’s standing on one or more specific skills, a level that is more actionable in the classroom.

Fourth, the review by Santos and Primi (2014) made clear that SEMS can be assessed following two conceptually different approaches. One is the trait or identity approach (Wiggins and Pincus, 1992; see John, 2021, for a review), which focuses on *typical* skilled behavior and asks “How do you typically or generally behave (or think or feel)?” We can think of this approach as measuring “lived skills,” that is the skill level at which an individual operates most of the time.

In contrast, other researchers have advocated an approach focused on capabilities or *maximal* behavior; here the interest is not on typical but on peak performance. In self-report form, students are asked “How well *can* you act (or think or feel) in a particular domain?”—in other words, self-efficacy. Bandura (1977) defined *self-efficacy* as students’ belief that they have the capability to organize and execute the actions required to manage future situations. Bandura (2006, p. 308) emphasized that self-efficacy items “should be phrased in terms of *can do* rather than *will do*. *Can* is a judgment of capability” (emphases in the original).

As so often, most researchers have adopted only one of these two approaches. Thus, we know little about how measures based on one approach correlate with measures based on the other. Bandura (2006) has argued that perceived self-efficacy is a major determinant of what people typically do (suggesting a substantial correlation) yet also emphasized that “the two constructs are conceptually and empirically separable” (p. 309).

It is entirely conceivable that some SEMS, like ‘trust’ or ‘responsibility,’ may be better conceptualized and measured from the typical performance (trait) approach, whereas other like ‘presentation skills’ may be better understood from an maximal behavior (self-efficacy) approach. How much students trust others on a day-to-day basis may well be a better predictor of their citizenship behavior, whereas how well they can present may be a better predictor of a student’s impact on an audience when they have to speak to a group. These kinds of hypotheses, however, that can be examined only if one has the two approaches to measure these skills (trait/identity versus self-efficacy) are represented in the same inventory.

Although we do not know how closely trait identity and self-efficacy measures are linked, the initial evidence is promising (Santos and Primi, 2014; Primi et al., 2016, 2019c): The three self-efficacy domains measured in children by Muris (2001) were substantially and differentially correlated with three of the trait identity measures included in the Socio-emotional Five: Social self-efficacy with Extraversion (Engaging with Others); Emotional Self-efficacy with Emotional Stability (Negative-Emotion Regulation); and Academic Self-Efficacy with Conscientiousness (Self-Management). Moreover, the items set initially developed for SENNA 1.0 included some self-efficacy items but only for three of the SEMS domains. Given that we wanted to take SEMS assessment seriously, we decided to represent both trait/identity and self-efficacy scales for all skills in the new measure. Thus, we will be able to test (a) whether the self-efficacy based SEMS scales analyzed separately show the expected Socio-emotional Five structure and (b) whether self-efficacy and trait-identity based scales will jointly define the same underlying factor structure.

Finally, given the anticipated heterogeneous and young respondent samples it is critically important to think about ways to reduce systematic error due to response styles that can compromise structural and predictive validity (Primi et al., 2019a,b,d, 2020). Soto et al. (2008) observed that psychometric and structural analyses of personality descriptive items of younger and less-educated samples rarely resemble the better structural validities found in adults and well-educated samples. These effects, discovered first in the United States, should be even more pronounced in the less educated youth in developing countries. Examining a large sample of youth aged 10 to 20 years, Soto et al. (2008) found that the underlying cause is large variability in how children and adolescents use the numerical rating scale to indicate how well a particular item describes themselves. Specifically, on a 1–5 rating scale, some youth will show high acquiescence and preferentially use the upper or right-hand side of the scale (i.e., use 4 and 5 far more often than 1 and 2) regardless of the content of the item. In contrast, others will show low acquiescence and preferentially use the lower or left-hand side of the scale (i.e., use 1 and 2 more often than 4 and 5). The first response bias is called high *acquiescence* because these youngsters agree more with items (yeah-saying), whereas the second response bias is named low acquiescence (nay-saying). Soto et al. (2008) showed that individual differences in acquiescence bias were most pronounced at age 10 to 12 and then decreased substantially (by 50%!) all the way to age 18, at which point they reached stable adult levels. Primi et al. (2019a) replicated these findings with public school students in Brazil and found that the acquiescence corrections substantially improved criterion validity. Having high-quality reverse (or false) keyed items is critical to assessing and controlling the effect of acquiescence. We therefore need carefully developed items that can be arranged like antonym pairs to measure not only high but also low levels of each of the SEMS dimensions in the new inventory.

### Assessing Social-Emotional Skills in Brazil: Development Steps Leading to the SENNA Inventory

Relying on our previous work for SENNA 1.0, a succinct review of the literature (e.g., John et al., 2008; Soto and John, 2017), and consultation of various educational stakeholders, we developed a blueprint of 18 SEMS, with tentative labels and short definitional descriptions, as shown in Table 2. SEMS are conceptually grouped under the Social-Emotional Big Five headers. These definitions formed the starting point to write and compile a large pool of 527 candidate items; 92 items came from the original SENNA 1.0 and 435 new items. This was an iterative emic process, involving multiple item writing and revision sessions, with input from research psychologists, education experts, and economists as well as former and current teachers. The item set included both positively and negatively keyed trait items, and also included items written specifically to reflect a self-efficacy rating perspective. Our goal was to construct short and homogeneous scales through item factor analysis with nine items each, including three positively keyed SEMS identity items, three negatively keyed identity items, and three SEMS self-efficacy statements—note that the self-efficacy items are, by definition, positively keyed (i.e., it doesn’t make sense to ask students how well they *can not* do something). The selection of items was done in a two-phase process relying on data from two different studies.

**TABLE 2:** Proposed socio-emotional skills domains, facets, and item examples for Senna-2.

Facet	Definition	Examples of items
**O: Open-mindedness: Interest and devotion to matters of the mind**		
1. Curiosity to learn	Able to muster interest in ideas and a passion for learning, understanding, and intellectual exploration; an *inquisitive* mind-set that facilitates critical thinking and problem solving (likes to think, play with ideas)	A lot of subjects awake my curiosity (identity +) I don’t have interest in finding out how things work (identity −) How well can you learn new things (self-efficacy)
2. Creative imagination	Is able to generate novel ways to think about or do things through experimenting, tinkering, learning from failure, insight, and vision (is original, comes up with new ideas)	I’m original, I have new ideas (identity +) I don’t have a lot of imagination (identity −) How well can you create and write stories (self-efficacy)
3. Artistic interest (appreciation of aesthetics):	Valuing, appreciating, and enjoying design, art, and beauty, which may be experienced or expressed in writing, visual and performing arts, music, and other forms of self-actualization (is fascinated by art, music, or literature)	I like artistic activities (identity +) I find art useless (identity −) How well can you create artistic things, like a poem (self-efficacy)
**C: Self-management (**or: **goal orientation, task performance)**		
1. Organization (orderliness):	Has organizational skills and meticulous attention to detail that are useful for planning and executing plans to reach longer-term goals (keeps their school things neat and tidy; not disorganized or messy)	I always keep my things organized (identity +) My things are messy (identity −) How well can you keep your school materials organized (self-efficacy)
2. Determination (goal striving, high standards):	Is able to set goals and high standards, motivate themselves, work very hard (in terms of time and effort), and apply themselves fully to the task, work, or project at hand. This is the pro-active side of C (I do more than what is expected of me; I do my work as well as I possibly can; vs. I only need to be in the average; I find it difficult to motivate myself to excel)	I’m a dedicated and hard-working student (identity +) I put little effort in my tasks (identity −) How well can you motivate yourself to always do your best (self-efficacy)
3. Focus (concentration):	Is able to focus attention and concentrate on the current task, and avoid distractions even while performing repetitive tasks (I manage to concentrate on things I do, vs. I don’t pay close attention during class and end up forgetting things)	Nothing distracts me once I start to work on a task (identity +) I deviate my attention easily (identity −) How well can you stay focused and not get lost when performing a task (self-efficacy)
4. Persistence (self-discipline):	Is able to overcome obstacles in order to reach important goals; “implement, persist, and finish.” The emphasis here is on completing tasks and finishing whatever one has undertaken, in contrast to procrastinating or giving up. Related concepts are grit, perseverance, and effortful control (I finish my work by the time I have planned to, vs. I leave everything until the last minute)	I never give up (identity +) I usually turn in work late. (identity −) How well can you apply yourself when preparing for a hard test (self-efficacy)
5. Responsibility (reliability, dependability):	Has self-management skills needed for doing one’s duty, meet commitments, act in reliable and consistent ways, and engender trustworthiness; this facet has a secondary link to A and should be important for predicting civic involvement and commitment (is reliable, can always be counted on)	I only make promises I know I’ll be able to fulfill (identity +) I usually forget about commitments that I have made. (identity −) How well can you keep your word, what you promised (self-efficacy)
**E: Engaging with others (vs. withdrawal and avoidance)**		
1. Social initiative:	Able to approach and connect with others, both friends and strangers, initiating, maintaining, and enjoying social contact and connections; skilled at teamwork, including expressive communication skills, such as public speaking skills (is outgoing, comfortable around people)	I’m uninhibited and I get along with others (identity +) I’m reserved, I keep to myself (Brazilian slang, don’t know how to translate properly) (identity −) How well can you make the first step to show that you like someone (self-efficacy)
2. Assertiveness (courage, finding your voice):	Able to speak up, voice opinions, needs, and feelings, and exert social influence; capacity to assert own will to accomplish goals in the face of opposition, such as speaking out, taking a stand, and confronting others if needed; courage (takes on leadership roles)	I usually give my opinion in group discussions (identity +) I don’t say anything when my classmates say something I don’t agree with. (identity −) How well can you ask your teachers for help when you have difficulties (self-efficacy)
3. Enthusiasm (energy; positive attitude):	Able to show passion and zest for life; to approach daily tasks with energy, excitement, and a positive attitude (is full of energy, shows enthusiasm)	I’m very happy and cheerful (identity +) I’m not a very excited person (identity −) How well can you cheer yourself up when you’re sad (self-efficacy)
**A: Amity (vs. enmity; tending and befriending others)**		
1. Empathy (compassionate caring):	Able to use empathy and perspective taking skills to understand the needs and feelings of others, act on that understanding with kindness and consideration of others, and investing in close relationships by helping and providing support and assistance, both material and emotional; is rewarding and easy to deal/live/work with (considerate and kind to everyone)	I care about what happens to others (identity +) I don’t care about other people’s feelings (identity −) How well can you understand what others are feeling (self-efficacy)
2. Respect for others (politeness):	Able to treat others with respect and politeness, the way oneself would like to be treated, according to notions of fairness, justice, and tolerance, and keeping aggressive and selfish impulses in check (is respectful; treats others with respect vs. breaking rules; known for defying teachers)	I respect authorities (teachers, principals, etc.) (identity +) I make threats to get what I want. (identity −) How well can you treat respectfully people you don’t like (self-efficacy)
3. Trust (forgiveness and appreciation of others):	Able to assume that others generally have good intentions and forgiving those that have done wrong; avoid being harsh and judgmental, giving people another chance (assumes the best about people)	I believe in the best in people (identity +) I feel it’s better not to trust anyone (identity −) How well can you trust people to watch over your things (self-efficacy)
4. Gratitude (humility):	Able to feel gratitude for what we have and humble about our abilities and status in the world, rather than thinking of oneself as better than others and deserving special treatment (I avoid calling attention to myself, vs. I put myself first because I am very special).	I don’t think I’m better than others (identity +) I think about myself first because I’m special (identity −) How well do you succeed in in being modest (self-efficacy)
**N: Negative-emotion regulation**		
1. Stress modulation:	Is effective in modulating anxiety and response to stress; untroubled by excessive worry and able to calmly solve problems (is relaxed, handles stress well)	After being scared, I calm down easily (identity +) I struggle with anxiety in difficult situations (identity −) How well can you deal with stress without worrying too much (self-efficacy)
2. Self-confidence (optimism):	Is able to feel satisfied with self and current life, think positive thoughts, and maintain optimistic expectations; anticipates success in actions undertaken; has a “can-do” mind-set; does not ruminate about failures, disappointments, or set-backs (feels secure, comfortable with self)	I’m happy and have few negative thoughts (identity +) I can’t stop thinking about negative things (identity −) How well can you stay in good spirits even when something bad happens to you (self-efficacy)
3. Tolerance of frustration (temper control):	Has effective strategies for regulating temper, anger, and irritation; able to maintain tranquility and equanimity in the face of frustrations; not moody or volatile (keeps their emotions and temper under control)	I stay calm and control my frustration (identity +) I get very angry and usually lose my temper (identity −) How well can you control your anger when other people make are annoying you (self-efficacy)

*Item examples were translated by the authors as literally as possible from the Brazilian Portuguese originals. The self-efficacy items are administered in a separate block from the trait identity items and are rated on a scale from 1 = not at all to 5 = very well.*

Our first study was conducted in 2014 and aimed to obtain a full inter-item covariance matrix across the 527 candidate items. Because we could not administer so many items to the same student, a Balanced Incomplete Block Design (BIB; van der Linden et al., 2004) was used to administer different subsets of items to different subsets of students, thus allowing us to estimate the full inter-item covariance matrix for every pair of items. Each student answered a booklet with a subset of 90 items on average, with a total of 24 paper-and-pencil booklets. The sample included 33,766 students from grades 5, 9, and 10 enrolled in public education in the State of Ceará, in the Northeast of Brazil. Students were randomly assigned to the 24 booklets. An average of 1,406 students answered each of the 24 booklets (min 1,268, max 1,427). We ran a series of exploratory factor and bi-factor analyses for the groups of items that designed to measure each of the intended 18 SEMS constructs. Our goal was to identify items that measured (a) the intended lower (or facet) level construct (e.g., Empathy) well and (b) showed good discrimination against both the other facets in the same domain (e.g., Respect) and the facets in the four other domains (e.g., Persistence in the Self-Management domain). After selecting preliminary candidate items for each facet, we ran exploratory factor analyses of candidate facet scales and inspected item-total correlations to further investigate convergent and discriminant validity. We selected those items that demonstrated a high loading on the general factor (from exploratory bi-factor analysis) and correlated more strongly with its intended facet scale than with other facets. As others have noticed in Western countries (e.g., Soto and John, 2017), results showed that it was particularly difficult to write items to measure low levels of Open-mindedness (e.g., lack of Intellectual Curiosity) and of high levels of Negative-Emotion Regulation; for example, items describing high levels of Self-confidence also tended to correlate with facets like Enthusiasm and Assertiveness (from Engaging with Others) and Persistence and Determination (from Self-Management). We retained the most promising items and wrote some additional candidate items for facets that were less well measured, resulting in a total of 306 items.

In a second study conducted in 2015, we analyzed these 306 items to select the final item set for SENNA. For this occasion, we developed a computerized web application that administered items in seven booklets to students using another BIB design. A sample of 5,485 high school students enrolled in public schools in the State of Ceará was randomly assigned to one out of the seven booklets (an average of 783 students answered each booklet), and each student answered a subset of 132 items. We followed the same method and rationale of the first study (internal structure analysis) to select the final set of 162 candidate items that composed SENNA. A more detailed description and materials of these two studies can be found in the SENNA manual (Primi et al., 2021).

To balance content across true keyed and false keyed items, and thus control acquiescence more systematically, we arranged the items on the trait identity scales into approximate antonym pairs. For example, a true-keyed identity item on the Curiosity to Learn facet scale (Open-mindedness domain) was “A lot of subjects awake my curiosity (identity +), whereas “I don’t have much interest in finding out how things work (identity −) was a reverse-keyed item (see Table 2 for more examples). A self-efficacy item example was “How well can you learn new things?” Thus, the self-efficacy items were all true keyed^5^ and their item content was developed separately from the identity items to highlight specific skill components associated with each of the 18 SEMS to be measured. The final SENNA measure thus included nine items per SEMS facet, specifically (a) six identity items (three positively and three negatively keyed items, forming three opposite pairs to permit us to compute and correct for acquiescence) and (b) three true-keyed self-efficacy items. Example items can be found in Table 2.

## Present Study

One limitation of our initial instrument development studies was that participants had always completed only subsets of our item pools. Here, we administered the full inventory to all participants at their schools, thus testing the feasibility of our assessment approach in the field. The present work first reports a joint factor analysis of the 18 positive identity, 18 negative identity, and 18 self-efficacy SEMS cluster scales to identify how these unfold into the Social-Emotional Big Five framework. We hypothesized that (a) the trait-identity clusters and self-efficacy clusters will load together on the expected SEMS factor, and (b) that acquiescence variance will affect the clarity of that structure, such that the structure obtained for the raw-score, uncorrected scales will be less clear, and adhere less well to the expected structure, than when the clusters are all corrected for individual differences in acquiescence. Soto et al. (2008) had found in Western youth aged 10–20 that the acquiescence effect was most pronounced for Agreeableness, and we examine whether that is also the case in our Brazilian public school students.

In addition, we examine whether separate analyses of (a) the 6-item identity clusters and (b) the 3-item self-efficacy clusters each shows the expected five-factor structure, testing our hypothesis that both approaches lead to measures that conform to the same underlying structure when acquiescence is controlled. Finally, we present the results of a joint principal component analysis of the acquiescence-corrected identity and self-efficacy clusters to test the hypothesis that the five-factor spaces for two approaches to SEMS measurement converge on a common model.

## Materials and Methods

### Participants

The sample included students from 234 cities and 501 public schools distributed across the entire State of São Paulo, and is the most comprehensive school-based assessment of SEMS attempted in Brazil so far. In total, 50,209 students completed the full 162-item SENNA item set via a dedicated web platform. Students were enrolled in grades 6 to 12, 52.7% were girls, and the average age was 14.9 years (SD = 2.1). All data were collected while students were participating in a reading program at their school.

### Design and Statistical Analysis

The present study aimed to test if a five-factor solution can account for the covariance matrix formed by of the three sets of 18 SENNA cluster scales described above (see examples for the three kinds of items in Table 2). For example, would the newly developed self-efficacy item clusters load along with the identity item clusters or form separate factors? We expected convergence across the trait-identity and self-efficacy approaches but not perfectly simple structure because several of the SEMS facet scales fall at the boundaries between the standard Big Five factors, like Self-Confidence (between Negative-Emotion Regulation and Engaging with Others) and Respect (between Amity and Self-Management). Finally, we wanted to investigate how acquiescence would affect the internal structure and whether these effects could be controlled estimating each respondent’s acquiescence tendency only from the (true and false keyed) trait identity items (as the self-efficacy items do not include any reverse-keyed items needed to compute acquiescence).

For each SEMS facet, three indicator variables were computed averaging scores on (a) three positively keyed identity items (‘identity +’), (b) three negatively (or false) keyed identity items (after reversing items so high scores always still reflect a high skill level; ‘identity −’), and (c) three positively keyed self-efficacy items, thus 18 SEMS constructs using three indicators with three items each, for a total of 54 short cluster scales. We then fitted a five-factor model with target rotation using Exploratory Structural Equation Modeling (ESEM) via MPLUS. We ran this analysis twice, one with the raw item scores and another with acquiescence corrected scores.

The identity scales of SENNA include 108 items (6 × 18) forming a balanced scale given that each facet has three positively and three negatively keyed items, or a total of 54 “antonym” pairs. We calculated an acquiescence index (ACQ) for each student, computing the average score across all 108 items, before reversing the negatively keyed items, per individual (see Soto et al., 2008, and Soto and John, 2017; for details on this procedure; Primi et al., 2020 for psychometric details and https://github.com/rprimi/noisecanceling for a R package to implement this method). If a student used the response scale in a fully symmetrical way, they would tend to have answer profiles such as 1–5, 2–4, 3–3, 4–2, or 5–1 to the two items in each semantic antonym, resulting in an ACQ score of 3, exactly at the mid-point of the 1–5 response scale labeled as:‘1’ (not at all like me), ‘2’ (little like me), ‘3’ (moderately like me), ‘4’ (a lot like me) and ‘5’ (completely like me)^6^.

If a student is more likely to agree regardless of the content of the items, the average across antonym pairs will be ACQ > 3, indicating elevated levels agreement (acquiescence bias). If the student is more likely to disagrees regardless of the content, ACQ < 3 indicating dis- acquiescence bias. This ACQ-index was used to correct all item responses, including the responses to the self-efficacy items, which do not include reverse-keyed items and thus ACQ cannot be estimated directly. We do that by subtracting the individual’s ACQ-index from each of their item scores. This procedure removes acquiescence variance from item scores (see more details in Primi et al., 2020).

## Results

### Overall Factor Structure Across Trait-Identity and Self-Efficacy Item Clusters

Table 3 shows the results of the ESEM internal structure analysis of the 54 indicators for the five-factor solution. The loadings on the left side of the table (columns four to eight) represent the final model when acquiescence variance was removed at the level of the individual respondent. The results were surprisingly clear: For 16 (out of 18) SEMS, all three indicators (positive identity, negative identity, and positive self-efficacy) had their highest loading on their intended social-emotional five-factor domain. Thus, quite consistently, positively and negatively keyed identity item clusters loaded together with their respective self-efficacy counterparts on the expected factors. There were two exceptions: the self-efficacy cluster for Assertiveness loaded more strongly on the Self-Management factor, whereas the self-efficacy cluster for Trust loaded more strongly on the Emotion Regulation factor. In fact, the other indicators of Trust did not clearly emerge as a facet of Amity, and were poorly represented in the overall factor solution.

**TABLE 3:** Factor loadings of Exploratory Structural Equation Modeling (ESEM) of 54 SEMS indicators, measured with either positively keyed or negatively keyed trait-identity items or self-efficacy items.

			Corrected for acquiescence					Raw scores				
Domain and facet	Item framing	Pole	O	C	E	A	N	O	C	E	A	N
**O: Openness**												
Artistic interest	Identity	–	**0.426**	0.079	0.046	**0.316**	–0.132	*0*.*229*	*0*.*250*	*0*.*261*	**0.468**	–0.092
	Identity	+	**0.589**	–0.062	–0.038	0.123	–0.016	**0.595**	0.037	0.009	–0.002	0.02
	Self-efficacy	+	**0.724**	0.000	–0.175	–0.033	0.128	**0.729**	0.042	–0.155	0.136	0.098
Creative imagination	Identity	–	**0.473**	0.029	0.170	0.106	–0.137	**0.338**	0.093	**0.305**	**0.438**	–0.112
	Identity	+	**0.703**	–0.040	0.150	–0.100	–0.050	**0.760**	–0.075	0.098	–0.039	–0.02
	Self-efficacy	+	**0.812**	0.021	–0.006	–0.110	0.121	**0.850**	–0.003	–0.022	0.106	0.089
Curiosity to learn	Identity	–	**0.351**	0.021	0.107	*0*.*263*	–0.177	0.175	0.165	*0*.*295*	**0.399**	–0.152
	Identity	+	**0.448**	0.066	0.143	0.183	–0.114	**0.483**	0.158	0.160	–0.184	–0.056
	Self-efficacy	+	**0.562**	0.169	0.049	0.070	0.123	**0.590**	*0*.*214*	0.071	–0.02	0.105
**C: Self-management**												
Determination	Identity	–	0.149	**0.360**	0.106	0.086	–0.132	0.037	**0.339**	0.203	**0.428**	–0.105
	Identity	+	0.018	**0.797**	–0.011	0.006	–0.079	0.149	**0.709**	–0.087	–0.114	–0.028
	Self-efficacy	+	0.168	**0.493**	0.148	0.069	0.132	*0*.*244*	**0.481**	0.103	–0.208	0.127
Focus	Identity	–	*0*.*205*	**0.524**	–0.103	–0.047	0.022	0.113	**0.431**	–0.042	**0.553**	0.039
	Identity	+	0.078	**0.560**	–0.046	0.032	0.046	0.194	**0.493**	–0.113	–0.143	0.093
	Self-efficacy	+	0.182	**0.574**	–0.110	–0.059	0.253	0.258	**0.508**	–0.191	–0.054	0.24
Organization	Identity	–	–0.149	**0.727**	–0.027	0.010	0.003	–0.182	**0.644**	–0.015	0.297	0.04
	Identity	+	–0.139	**0.867**	–0.031	–0.014	–0.020	0.007	**0.752**	–0.131	–0.157	0.026
	Self-efficacy	+	–0.051	**0.750**	–0.063	–0.047	0.183	0.047	**0.669**	–0.161	–0.122	0.175
Persistence	Identity	–	0.013	**0.716**	0.016	0.103	–0.191	–0.052	**0.700**	0.086	0.336	–0.144
	Identity	+	0.007	**0.673**	0.124	0.033	–0.046	0.145	**0.585**	0.033	−*0*.*236*	0.01
	Self-efficacy	+	0.165	**0.680**	–0.074	–0.063	0.099	*0*.*253*	**0.620**	–0.15	–0.043	0.079
Responsibility	Identity	–	–0.008	**0.459**	0.173	*0*.*214*	–0.126	–0.108	**0.493**	*0*.*280*	**0.299**	–0.075
	Identity	+	–0.042	**0.530**	0.123	*0*.*265*	–0.100	0.039	**0.599**	0.128	−*0*.*283*	–0.033
	Self-efficacy	+	0.029	**0.456**	0.037	*0*.*268*	0.085	0.049	**0.583**	0.080	−*0*.*211*	0.088
**Engaging with others**												
Enthusiasm	Identity	–	0.079	–0.074	**0.534**	0.052	–0.041	0.007	–0.100	**0.575**	0.204	–0.021
	Identity	+	–0.017	0.063	**0.584**	0.060	*0*.*221*	0.120	–0.025	**0.467**	–0.360	0.264
	Self-efficacy	+	0.080	*0*.*236*	**0.300**	0.137	**0.322**	0.142	*0*.*232*	**0.279**	–0.241	**0.329**
Assertiveness	Identity	–	0.195	0.126	**0.445**	−*0*.*207*	0.01	0.158	–0.052	**0.400**	**0.327**	–0.004
	Identity	+	*0*.*261*	0.139	**0.351**	–0.188	–0.123	**0.402**	0.004	0.192	−*0*.*288*	–0.096
	Self-efficacy	+	0.189	**0.381**	0.191	–0.130	0.179	*0*.*288*	0.255	0.093	–0.088	0.158
Social initiative	Identity	–	0.052	–0.181	**0.607**	–0.073	0.023	0.015	−*0*.*263*	**0.587**	0.086	0.013
	Identity	+	–0.050	–0.078	**0.649**	*0*.*223*	0.014	0.055	–0.045	**0.605**	−**0.506**	0.051
	Self-efficacy	+	0.059	0.072	**0.316**	0.050	*0*.*259*	0.132	0.052	**0.252**	−**0.333**	*0*.*243*
**A: Amity**												
Empathy	Identity	–	0.148	0.074	*0*.*215*	**0.451**	–0.141	–0.021	*0*.*299*	**0.423**	0.255	–0.078
	Identity	+	0.053	–0.022	*0*.*217*	**0.540**	–0.013	0.063	*0*.*243*	**0.309**	−**0.401**	0.051
	Self-efficacy	+	*0*.*245*	0.039	0.184	**0.388**	–0.006	*0*.*237*	*0*.*263*	*0*.*279*	−**0.321**	–0.011
Gratitude	Identity	–	0.036	0.064	–0.109	**0.615**	–0.083	–0.184	**0.375**	0.185	**0.330**	–0.034
	Identity	+	0.050	–0.093	–0.199	**0.346**	–0.045	0.041	**0.129**	–0.101	−*0*.*202*	0.010
	Self-efficacy	+	0.187	0.124	–0.175	**0.318**	0.249	0.161	**0.313**	–0.091	−*0*.*135*	*0*.*248*
Respect	Identity	–	0.031	*0*.*299*	−*0*.*216*	**0.457**	0.138	–0.153	**0.492**	0.019	**0.438**	0.184
	Identity	+	–0.016	0.366	–0.025	**0.508**	–0.017	–0.013	**0.593**	0.107	–0.158	0.056
	Self-efficacy	+	0.136	0.166	–0.168	**0.437**	**0.353**	0.084	**0.402**	–0.043	–0.087	**0.375**
Trust	Identity	–	–0.011	–0.007	0.128	0.087	*0*.*267*	–0.126	–0.011	0.201	**0.381**	*0*.*266*
	Identity	+	–0.002	–0.117	*0*.*202*	**0.326**	0.187	0.040	0.022	0.220	−**0.341**	*0*.*232*
	Self-efficacy	+	0.069	–0.129	0.123	0.173	**0.347**	0.088	–0.055	0.124	−*0*.*229*	0.347
**N: Negative-emotion regulation**												
Frustration tolerance	Identity	–	0.046	–0.041	–0.023	0.176	**0.586**	–0.108	–0.033	0.094	**0.545**	**0.640**
	Identity	+	–0.036	–0.060	–0.081	0.137	**0.527**	0.000	–0.051	–0.101	–0.057	**0.601**
	Self-efficacy	+	0.071	–0.074	–0.124	*0*.*205*	**0.747**	0.031	–0.004	–0.094	0.027	**0.789**
Stress modulation	Identity	–	0.031	0.085	0.193	–0.189	**0.444**	–0.031	–0.113	0.164	**0.484**	**0.434**
	Identity	+	0.046	0.102	0.214	–0.033	**0.397**	0.166	–0.003	0.086	−*0*.*227*	**0.428**
	Self-efficacy	+	0.110	0.034	–0.028	0.033	**0.739**	0.128	–0.001	–0.081	–0.035	**0.751**
Self-confidence	Identity	–	–0.127	*0*.*238*	*0*.*294*	0.009	**0.349**	–0.183	0.095	**0.306**	**0.403**	**0.354**
	Identity	+	–0.068	0.134	*0*.*291*	0.126	**0.300**	0.041	0.090	*0*.*217*	−*0*.*256*	**0.368**
	Self-efficacy	+	0.068	0.169	0.254	0.090	**0.496**	0.123	0.137	*0*.*206*	–0.21	**0.502**

*Loadings higher then *r* > 0.30 in bold. Loadings higher than *r* > 0.20 in italics. Fit indices were: (a) Raw scores χ^2^ = 55,271.8, df = 1,171, BIC = 1,489,415, CFI = 0.83, TLI = 0.79, RMSEA = 0.06, SRMR = 0.04. (b) Corrected for acquiescence: χ^2^ = 78,888.0, df = 1,171, BIC = 1,437,677, CFI = 0.74, TLI = 0.68, RMSEA = 0.07, SRMR = 0.04.*

As expected, some secondary loadings were observed: (a) Responsibility, a facet of Self-Management, also had secondary loadings on Amity across all indicators. This skill is conceptualized as the most interpersonal aspect of Self-Management and implies a commitment to others, thus associating it also with Amity; (b) The self-efficacy indicator of Enthusiasm, a facet of Engagement with Others, also loaded on Emotion Regulation because its items refer to experiencing energy and positive emotions in stressful situations; (c) The self-efficacy indictor of Respect, a facet of Amity, has a secondary loading on Emotion Regulation as items refer to the regulation of negative behavior and impulses (e.g., suspicion) in interpersonal situations; (d) Frustration tolerance, a facet of Emotion Regulation, also had a secondary loading on Amity because items refer to the regulation of anger and irritability in social situations that connects to caring with others; (e) Finally, all indicators of Self Confidence, a facet of Negative-Emotion Regulation, had secondary loadings on Engagement with others. These items refer to positive confidence in the self that is a necessary condition to reach out to others.

### Effects of Acquiescence on Internal Structure

The second focus of our analyses was examining the effect of acquiescence correction on the internal structure. The acquiescence index had a mean of *M* = 2.95 (close to the expected 3.0, the mid-point of the rating scale); however, the standard deviation (SD) was 0.37, indicating substantial individual differences in scale usage across the students and thus deviation from the expected score of 3.0. Thus, we expected acquiescence would have a salient effect.

We computed a similar ESEM model but now with the uncorrected raw scores and loadings of indicators are reported on the right-hand side of Table 3 (in columns 9 to 13). An inspection of these columns shows that in every broad SEMS domain, a substantively higher number of indicator scales had their primary loading on another factor than intended. This was mainly due to the negatively keyed identity indicators of several SEMS, which had their primary loading on the fourth factor. Overall, in this analysis, the Amity domain was not recovered, due to a strong influence of acquiescence in the covariance matrix. The fourth factor extracted in this analysis seems to reflect acquiescence variance because it has negative items with positive loadings contrasted with positive items with negative loadings.

To quantify these observations, we compared the ESEM results from the raw and the acquiescence-corrected SEMS indicators further using a novel variant of factor congruence analysis. To define a theory-based, idealized target matrix of loadings, we created a vector of 54 numbers for each of the five expected domains, one for each indicator variable, with perfect theoretical loadings of 1.0 only on the one intended domain and zero on all the other four domains (thus, somewhat unrealistically, not allowing any of the known secondary loadings). We then correlated this idealized target matrix with the observed loadings of the 18 indicators estimated by the ESEM model, separately for the raw and for the acquiescence-corrected scores. These congruence coefficients are presented in Table 4.

**TABLE 4:** Conceptual congruency coefficients: Correlations of the empirically observed loadings on the five factors with *a priori* theoretical (ideal) loadings (1 and 0) for (a) scores corrected for acquiescence (upper half) and (b) uncorrected raw scores (lower half).

	Theoretical factor loadings				
Empirical factor loadings	O	C	E	A	N
**Corrected for acquiescence scores**					
O	**0.89**	0.08	0.15	0.14	0.02
C	0.04	**0.92**	0.09	0.09	0.08
E	0.09	0.04	**0.78**	0.03	0.19
A	0.16	0.13	–0.01	**0.79**	0.11
N	–0.04	0.02	0.15	0.19	**0.80**
**Raw scores**					
O	**0.80**	0.16	0.22	0.03	0.03
C	0.11	**0.85**	0.01	0.34	0.01
E	0.20	0.00	**0.67**	0.27	0.15
(A)	0.21	0.04	–0.19	–0.06	0.11
N	–0.02	0.07	0.16	0.24	**0.83**

*The numbers shown in bold font indicate the highest congruence coefficient for each empirical factor and thus the best match between the empirically obtained factor and the theoretically expected factor. In the raw-score analyses (in the lower half of the table), the expected A factor was not identified clearly as a separate factor.*

The upper half of Table 4 shows the congruence coefficients for the acquiescence-corrected ESEM model, whereas the lower half shows the congruence coefficients for the raw-score model. Each cell shows the congruence coefficient for comparing the empirical loadings (rows) to the idealized theoretical loadings (columns). We calculated these coefficients for all pairwise combinations. If structures were identical, then the values on the diagonal would all be 1.0 and the off-diagonal values would be zero.

When we consider the data corrected for acquiescence, the loadings were much closer to this ideal. The five congruence coefficients on the diagonal averaged 0.85 and even the lowest was 0.78. In contrast, for the raw scores, the diagonal congruence coefficients averaged only 0.62. Moreover, the off-diagonal coefficients were much closer to zero for the acquiescence-corrected ESEM model. In other words, the loading pattern for the raw scores was a far less clear representation of the social-emotional five-factor domains than the acquiescence-corrected one.

As expected on the basis of Western research, acquiescence affected the SEMS indicators in the Amity domain most strongly. Looking at the congruency of the empirical factor in row A in Table 4, there was no similarity of empirical loadings with the theoretically expected values for A (i.e., column A: *r* = −0.06) or with any other domain (O: *r* = 0.21, C: *r* = 0.04, E: *r* = −0.19, N: *r* = 0.11). When we look at column A (which represents the vector of theoretically expected perfect loadings of 1s and 0s), we see only small correlations with empirical loadings for factors representing C (*r* = 0.34), E (*r* = 0.27), and N (*r* = 0.25).

### Structure of Identity Versus Self-Efficacy Indicators

Finally, we tested whether the newly devised self-efficacy scales would be well represented within the now familiar five-factor structure. We conducted separate principal component analyses of (a) the 18 SENNA identity indicators (now combining their true and false-keyed items into one 6-item scale) and (b) the self-efficacy indicators (three items each) after all indicators had been corrected for acquiescence. We used principal components because we wanted a model with no constraints in loadings. As can be observed in Table 5, the self-efficacy scales grouped as expected according to the five domains, and so did the identity scales. Some of the self-efficacy scales, however, showed secondary loadings on the Self-management factor: Curiosity to learn (O), Assertiveness (E), Gratitude (A), and Self-Confidence (N) had substantive loadings on Self-Management.

**TABLE 5:** Separate factor structures for the trait-identity item clusters (Id) and for the self-efficacy (SE) item clusters: Standardized loadings from a principal component analysis after controlling for acquiescence and communality (h^2^) to indicate total variance explained.

	O		C		E		A		N		h^2^	
						
	Id	SE	Id	SE	Id	SE	Id	SE	Id	SE	Id	SE
O1: Creative imagination	**0.77**	**0.82**	0.21	0.23	0.14	0.15	–0.03	0.11	0.17	0.14	0.68	0.79
O2: Curiosity to learn	**0.75**	**0.66**	0.20	**0.38**	0.11	0.21	0.17	0.13	0.01	0.17	0.64	0.67
O3: Artistic interest	**0.63**	**0.83**	0.25	0.20	0.08	–0.03	0.27	0.18	0.17	0.16	0.56	0.78
C1: Persistence	0.21	**0.37**	**0.83**	**0.75**	0.08	0.10	0.07	0.04	0.08	0.12	0.74	0.72
C2: Determination	0.26	0.28	**0.77**	**0.67**	0.08	**0.31**	0.06	0.11	0.09	0.13	0.67	0.66
C3: Organization	0.02	0.17	**0.81**	**0.74**	–0.02	0.13	0.05	0.04	0.18	0.21	0.68	0.64
C4: Focus	0.28	0.34	**0.70**	**0.69**	–0.10	0.08	0.05	0.03	**0.30**	**0.30**	0.66	0.69
C5: Responsibility	0.16	0.01	**0.71**	**0.72**	0.17	0.05	0.09	**0.37**	0.06	0.14	0.57	0.67
E1: Social initiative	0.09	0.05	–0.09	0.04	**0.84**	**0.77**	–0.08	0.22	–0.01	0.08	0.73	0.66
E2: Enthusiasm	0.18	0.17	0.08	0.38	**0.77**	**0.52**	–0.03	0.13	0.19	0.33	0.67	0.56
E3: Assertiveness	**0.45**	**0.33**	0.19	**0.47**	**0.42**	**0.38**	−**0.40**	–0.07	0.07	0.11	0.57	0.49
A1: Empathy	0.23	0.18	0.29	0.26	**0.49**	0.27	**0.56**	**0.72**	–0.03	–0.11	0.68	0.70
A2: Respect	0.10	0.17	**0.59**	**0.47**	–0.05	–0.05	**0.49**	**0.38**	0.28	**0.50**	0.68	0.65
A3: Trust	–0.05	0.12	0.12	–0.07	**0.42**	0.20	**0.44**	**0.57**	**0.38**	**0.31**	0.53	0.48
A4: Gratitude	0.18	0.14	0.09	**0.45**	–0.16	–0.19	**0.67**	**0.43**	–0.01	**0.37**	0.51	0.58
N1: Frustration tolerance	0.12	0.13	0.14	0.17	–0.07	0.09	0.19	0.10	**0.81**	**0.86**	0.73	0.80
N2: Stress modulation	0.18	0.19	0.17	0.24	0.12	0.22	–0.19	0.03	**0.77**	**0.79**	0.70	0.77
N3: Self-confidence	0.04	0.15	**0.31**	**0.35**	**0.35**	**0.48**	0.06	0.11	**0.62**	**0.52**	0.61	0.65

*Loadings higher then r > 0.30 in bold.*

When analyzed together with the identity items, self-efficacy scales still had their highest loadings on the five factors along with their corresponding identity scales (see Table 6). SEMS identity and self-efficacy items hence seem to function as indicators of the same underlying latent social-emotional five factors.

**TABLE 6:** Joint principal component analysis of person-centered identity and self-efficacy scales: Standardized loadings and communality (h^2^) to indicate total variance explained.

	O	C	E	A	N	h^2^
ID-O1: Creative imagination	0.75	0.16	0.22	0.03	0.08	0.64
ID-O2: Intellectual curiosity	**0.62**	0.20	0.15	0.19	–0.04	0.48
ID-O3: Aesthetic interest	**0.69**	0.20	0.04	0.25	0.11	0.59
SE-O1: Creative imagination	**0.76**	0.26	0.12	0.01	0.25	0.72
SE-O2: Intellectual curiosity	**0.64**	**0.37**	0.14	0.08	0.27	0.64
SE-O3: Aesthetic interest	**0.72**	0.24	–0.04	0.10	0.23	0.64
ID-C1: Persistence	0.23	**0.77**	0.11	0.11	0.05	0.67
ID-C2: Determination	0.28	**0.71**	0.10	0.11	0.05	0.61
ID-C3: Organization	0.09	**0.77**	0.00	0.04	0.16	0.62
ID-C4: Focus	0.32	**0.66**	–0.04	0.05	0.23	0.59
ID-C5: Responsibility	0.11	**0.68**	0.18	0.23	0.01	0.55
SE-C1: Persistence	0.28	**0.73**	0.03	0.06	0.21	0.66
SE-C2: Determination	0.28	**0.64**	0.24	0.08	0.22	0.60
SE-C3: Organization	0.09	**0.75**	0.03	0.04	0.28	0.65
SE-C4: Focus	0.27	**0.67**	–0.01	0.04	**0.37**	0.66
SE-C5: Responsibility	0.05	**0.66**	0.10	0.27	0.17	0.55
ID-E1: Social initiative	0.03	–0.12	**0.78**	0.17	–0.06	0.65
ID-E2: Assertiveness	0.32	0.18	**0.57**	–0.18	–0.03	0.50
ID-E3: Enthusiasm	0.15	0.04	**0.73**	0.16	0.10	0.59
SE-E1: Social initiative	0.03	0.12	**0.46**	0.07	0.24	0.29
SE-E2: Assertiveness	0.26	**0.44**	**0.33**	–0.07	0.23	0.43
SE-E3: Enthusiasm	0.15	**0.36**	**0.41**	0.14	**0.42**	0.51
ID-A2: Empathy	0.22	0.23	0.30	**0.65**	–0.05	0.62
ID-A1: Respect	0.18	**0.56**	–0.15	**0.43**	0.26	0.61
ID-A3: Trust	0.05	0.04	0.27	**0.57**	**0.30**	0.49
ID-A4: Gratitude	0.09	0.15	−**0.30**	**0.52**	–0.02	0.39
SE-A2: Empathy	0.23	0.26	0.29	**0.46**	–0.03	0.42
SE-A1: Respect	0.19	**0.43**	–0.11	**0.41**	**0.46**	0.61
SE-A3: Trust	0.01	–0.01	0.24	**0.53**	**0.33**	0.45
SE-A4: Gratitude	0.18	0.36	–0.16	**0.36**	**0.33**	0.43
ID-N1: Frustration tolerance	0.13	0.13	–0.04	0.14	**0.73**	0.58
ID-N2: Stress modulation	0.15	0.18	0.28	–0.11	**0.61**	0.52
ID-N3: Self-confidence	0.06	0.29	**0.37**	0.11	**0.47**	0.46
SE-N1: Frustration tolerance	0.11	0.16	–0.05	0.17	**0.80**	0.70
SE-N2: Stress modulation	0.14	0.23	0.07	0.06	**0.78**	0.70
SE-N2: Self-confidence	0.13	**0.34**	**0.34**	0.08	**0.59**	0.60

## Discussion

This paper described the developmental history of SENNA and provided new psychometric information on its internal structure obtained from a large sample of 50,000 Brazilian students enrolled in public school grades 6 to 12, spread across the entire State of São Paulo. The SENNA inventory was designed as an instrument assessing 18 different skills, each operationalized by nine items that represent three types of items: three positively keyed identity items and three negatively keyed identity items, complemented with three (always positively keyed) self-efficacy items, totaling a set of 162 items. Individual skills were assumed to group into the higher-order structure of the social-emotional Big Five, labeled as Engaging with Others, Amity, Self-management, Emotional Regulation, and Open-mindedness. Given its youth target group is as young as 11 years old (grade 6), SENNA was also equipped to correct systematically for individual differences in acquiescence which are known to have particularly strong biasing effects from ages 10 to 13 (Soto et al., 2008).

### The Social-Emotional Big Five

Our results showed convincing evidence that the 18 SEMS measured here aligned within the social-emotional Big Five structure, both when analyzing the identity and self-efficacy scales together and also when they were analyzed separately, with one critical condition: in samples of youth like our’s, with children as young as 11 years old, individual differences in acquiescence have to be corrected. There were some expected secondary loadings that have also been observed in Western samples (e.g., Soto et al., 2008; Soto and John, 2017). The results for the Trust skill in this study were less clear, and this scale was more difficult to position uniquely in the social-emotional skill space. Overall, these findings underscore that the social-emotional Big Five is also a useful framework to organize SEMS in a non-WEIRD culture like Brazil (John et al., 2008; Kyllonen et al., 2014; Lipnevich et al., 2016; Abrahams et al., 2019; Primi et al., 2019c).

This work further provided strong evidence that students from grades 6 to 12 are able to provide reliable and valid descriptions on identity and self-efficacy scales to assess a broad variety of SEMS, even when data are collected in the course of large-scale assessments. The instrument hence meets all requirements that were previously listed, including being broadly applicable, at a low cost (in time and financially), and to be completed independently by students. The SENNA inventory in its present form, is a significant step forward compared to its predecessor (SENNA 1.0; Primi et al., 2016) including (a) scales to assess five domains and 18 specific SEMS, (b) representing both identity and self-efficacy items to represent the constructs, and (c) an effective method to deal with acquiescence bias in responding.

### Acquiescence Matters

Furthermore, this study demonstrated the importance of having a proper method to control for acquiescence variance, that is a main problem in large-scale assessment where participants are often younger, represent various backgrounds, are not necessarily motivated or paying attention during the entire assessment, and where some participants may demonstrate answering tendencies such as yes- or no-saying. Low motivation to complete assessments may produce inconsistent agreement and disagreements. In the literature, this is called Insufficient Effort Responding (Niessen et al., 2016). Since acquiescence varies across students, it may influence inter-item correlations confounding the correlations that we expect to be caused by the latent dimensions we are trying to measure. This systematic confounding suppresses correlations of items of opposite poles and inflates correlations between items assessing the same pole (++ and −−, Maydeu-Olivares and Steenkamp, 2018; Mirowsky and Ross, 1991; Primi et al., 2020; Primi et al., 2019b).

In this sample, as well as in our previous work (Primi et al., 2016), we observe sizable acquiescence variance. This influence usually produces two significant factors grouping positive and negative items. As Ten Berge (1999) points out, sometimes we can find an acquiescence factor having all items - positive and negative before reversing - loading positively on a factor (or reversed negative scales with positive loadings conflated with positive facet scales with positive loadings on a factor as was in our case) that can be interpreted as an acquiescence factor. In sum, acquiescence is a “ghost” cofounder that needs to be controlled before we can run item factor analysis. In our study, the Amity factor was difficult to recover, except when we controlled for acquiescence.

### Trait-Identity and Self-Efficacy

While developing SENNA, we systematically included both trait-identity and self-efficacy items to assess all SEMS, to be in a position to systematically examine their relative contributions to assessing SEMS and investigate their validities to predict education outcomes of interest. Contributions and validities could be systematically different for methods (identity versus self-efficacy), but could also depend on the SEMS considered (see our example of trust and presentation skills in the introduction). As it stands now, and relying on the findings of the current study, we aggregate scores on identity and self-efficacy scales and take these aggregates as an index of a particular SEMS. This practice may have to be amended in the future, when new evidence would become available, demonstrating differential predictive validities favoring one over another measurement perspective (identity relative to self-efficacy). Although both assessment perspectives can be distinguished conceptually, it is not clear at the moment whether identity and self-efficacy items can be empirically distinguished well.

Moreover, the combination of positively- and negatively keyed identity items helped to correct for acquiescence. Scales composed exclusively of self-efficacy items miss negatively keyed items (low-skill items) and hence do not allow for the identification and correction for acquiescence bias. The combination of identity items (both positively and negatively keyed) together with self-efficacy items also enables the researcher to correct the self-efficacy items for acquiescence bias.

### Practical Contributions

SENNA was primarily designed for implementation in education practice and policy making, but it should also serve fundamental and applied research purposes. We provided new evidence in this paper supporting its use and highlighting some of its research opportunities. Use of the inventory, its scales and its reports will help education in Brazil to use a common vocabulary to talk about SEMS development among students, teachers, parents, directors and policy makers. In addition, it creates an opportunity to develop evidence-based actions to be implemented in the classroom but also for policy-making. Intensive policy debates started in many countries, including Brazil, to represent SEMS learning in the educational curriculum (OECD, 2015). One significant initiative in Brazil is the Brazilian Common Core for Teaching Fundamentals^7^, referring to global competencies that tap into various combinations of more foundational SEMS skills. SENNA results can provide input for this debate.

From a research perspective, the demonstration of the relationships between SEMS at school and outcomes across life will be important. Further examining the conceptual distinction between identity versus self-efficacy measurement perspectives will be additional avenues of research. Finally, the supplementary confirmation of the social-emotional Big Five framework as a model to structure SEMS also opens new perspectives to think about the construction of more formative assessment tools that can be directly used within classrooms to inform the learning of SEMS in education (Pancorbo et al., 2020).

### Limitations

The present work has a number of strengths, such as relying on a large sample systematically sampled from public schools in a culture for which the instrument was designed, and providing a nuanced approach to represent diverse content and multiple measurement angles to assess SEMS. There are, however, also a number of limitations, that readers and potential users need to take into account. First, the present data are self-reports susceptible to a range of biases, beyond acquiescence. Second, at the present stage, SENNA is not designed to be used in summative assessment contexts, where the result of an assessment has important consequential outcomes, such as investing in new programs or providing extra support for teachers.

Finally, evidence for external and criterion evidence in scholastic contexts is needed. One paper (Primi et al., 2019a) has already shown that the broad SENNA domain scales are related to students’ objective test scores, with the Self-Management and Open-Mindedness domain scores showing the expected strongest validity coefficients. Future research needs to test the reasonable hypothesis that going from this broad level to the lower level of specific SEMS will further improve the prediction of important scholastic and life outcomes in public school students.

## Author’s Note

This article is part of a research program on socio-emotional skills sponsored by the Ayrton Senna Foundation. RP also receives a scholarship from the National Council on Scientific and Technological Development (CNPq) and funding from São Paulo Research Foundation (FAPESP). Coordenação de Aperfeiçoamento de Pessoal de Nível Superior – Brasil (CAPES) PVEX – 88881.337381/2019-01.

## Data Availability Statement

The raw data supporting the conclusions of this article will be made available by the authors, without undue reservation.

## Ethics Statement

Studies involving human participants were reviewed and approved by Universidade São Francisco. Written informed consent for participation was not required in accordance with the local legislation and institutional requirements. Data collected for this study were part of student’s regular school activities involving minimal risk. We received approval from the educational secretariat to collect data as part of these activities.

## Author Contributions

RP, DS, OJ, and FD contributed to the conception and design of the study. RP analyzed the data and wrote the first draft of the manuscript. OJ, FD, and DS revised and wrote substantial sessions of the manuscript. All authors contributed to manuscript revision, read, and approved the submitted version.

## Conflict of Interest

The authors declare that the research was conducted in the absence of any commercial or financial relationships that could be construed as a potential conflict of interest.

## Publisher’s Note

All claims expressed in this article are solely those of the authors and do not necessarily represent those of their affiliated organizations, or those of the publisher, the editors and the reviewers. Any product that may be evaluated in this article, or claim that may be made by its manufacturer, is not guaranteed or endorsed by the publisher.

## Footnotes

1
https://www.vuca-world.org

2
http://exploresel.gse.harvard.edu

3
https://www.oecd.org/pisa/publications/

4
https://www.oecd.org/education/ceri/social-emotional-skills-study/

5 We wrote only positively keyed self-efficacy items because it is strange to ask students to rate “how well they cannot do something.” Given that acquiescence is considered a more general response tendency, we used the acquiescence index computed on the identity items to also correct the raw scores of the self-efficacy items.

6 Note that these labels match trait identity items asking students to think about “*How do you behave/feel and think in most situations.*” For self-efficacy items asking students “*How well can you do.*” we used: ‘1’ (nothing capable), ‘2’ (little capable), ‘3’ (moderately capable), ‘4’ (very capable) and ‘5’ (totally capable).

7
http://basenacionalcomum.mec.gov.br/
